# Efficacy of RCI001 as a therapeutic candidate of dry eye disease in a modified mixed dry eye model

**DOI:** 10.1186/s40662-024-00388-z

**Published:** 2024-06-01

**Authors:** Young-ho Jung, Young Ah Ku, Jayoon Moon, Seunghoon Kim, Jin Suk Ryu, Chang Ho Yoon, Myung Hee Chung, Yong Ho Kim, Mee Kum Kim, Dong Hyun Kim

**Affiliations:** 1 Department of Ophthalmology, Seoul National University College of Medicine, Seoul, Republic of Korea; 2 Department of Ophthalmology, Seoul National University College of Medicine, Seoul, Republic of Korea; 3 Laboratory of Ocular Regenerative Medicine and Immunology, Biomedical Research Institute, Seoul National University Hospital, Seoul, Republic of Korea; 4 RudaCure Co., Ltd, Incheon, Republic of Korea; 5 Department of Pharmacology, Seoul National University College of Medicine, Seoul, Republic of Korea; 6 Department of Ophthalmology, Saevit Eye Hospital, Goyang, Republic of Korea; 7 Gachon Pain Center and Department of Physiology, Gachon University College of Medicine, Incheon, Korea; 8 Department of Ophthalmology, Korea University College of Medicine, Seoul, Republic of Korea

**Keywords:** RCI001, Dry eye disease, Inflammation, Oxidative stress, Therapeutics

## Abstract

**Background:**

To evaluate the therapeutic effects of topical RCI001 (RCI) and compare its efficacy with that of 1% prednisolone acetate (PDE) and 5% Lifitegrast in a modified mixed dry eye disease (DED) model.

**Methods:**

The environmental DED model was induced in BALB/c mice in a dry chamber with scopolamine. The eyes of the mice were treated topically with phosphate buffered saline (PBS), PDE, Lifitegrast or RCI twice daily for 1 week. Ocular surface staining (OSS), tear secretion, inflammatory cytokines in the ocular surface and lacrimal gland, and immunofluorescence staining in the conjunctiva and cornea(CC) were assessed.

**Results:**

The RCI group demonstrated better improvement of OSS and tear secretion than the PBS group (OSS, PBS: 13.0 ± 1.6, RCI: 9.4 ± 3.0; tear secretion, PBS: 5.0 ± 0.4 mm, RCI: 7.0 ± 0.3 mm, each *P* < 0.001) and better clinical efficacy than PDE and Lifitegrast groups on Day 7 (improvement rate of OSS, RCI: 32.45%, Lifitegrast: 13.13%, PDE: 12.25%). The RCI group resulted in significantly lower expression of oxidative stress markers in the CC than the PBS group (4-HNE, NOX2, and NOX4 in the conjunctiva; NOX2 in the cornea, each *P* < 0.05). However, the PDE and Lifitegrast groups did not show significant differences compared with the PBS group. There were no significant differences of inflammatory cytokines in the ocular surface and lacrimal gland between all groups.

**Conclusion:**

Topical RCI001 showed excellent therapeutic effects in environmental DED models by stimulating tear secretion, modulating oxidative stress and improving corneal epithelial healing compared to 1% PDE and 5% Lifitegrast.

**Supplementary Information:**

The online version contains supplementary material available at 10.1186/s40662-024-00388-z.

## Background

Dry eye disease (DED) is a representative ocular surface disease (OSD) and is defined as a multifactorial disease of the ocular surface characterized by a loss of tear film homeostasis accompanied by ocular symptoms [1]. The prevalence of DED based on symptoms and signs ranges from 5% to 50% worldwide [2]. The overall annual cost for the management of DED was estimated to be 3.84 billion USD and annual cost per patient with DED was estimated to be 11,302 USD in the United States [2, 3]. Furthermore, modern socioeconomic lifestyles and environmental conditions such as medication, cosmetics, digital devices, pollution, and low humidity can also aggravate DED, especially hyper evaporative status and may intensify its overall prevalence [2, 4].

DED is mainly caused by tear hyperosmolarity and tear film instability owing to desiccating stress [5]. Consequently, it causes stress and damage of the ocular surface epithelium, which finally triggers complex inflammatory cascades of the innate (epithelial cells, dendritic cells, neutrophils, and macrophages; acute response) and adaptive (T cells, B cells; chronic response) immune responses [5, 6]. The vicious cycle of inflammation is regarded as a core aggravating factor in DED with the interaction between the sensory neurons of the ocular surface and local immune system disrupting ocular surface homeostasis [7, 8]. Therefore, anti-inflammatory drugs such as corticosteroids, cyclosporine A, and Lifitegrast, have been commonly applied to treat DED [7]. Diquafosol and rebamipide are also used to reduce desiccating stress on the ocular surface by stimulating tear fluid or mucin secretion [7, 9, 10]. Topical corticosteroids are potent inhibitors of multiple inflammatory mediators that can suppress myeloid cell infiltration, maturation of antigen-presenting cells, and expression of matrix metalloproteinases (MMPs), chemokines, and inflammatory cytokines such as interleukin (IL)-1 and tumor necrosis factor (TNF)-α [7, 8]. Nonetheless, long-term corticosteroid usage induces elevated intraocular pressure, cataract formation, and secondary infection [7]. Although T cell inhibitors such as cyclosporin A and Lifitegrast are known to treat DED effectively, these agents are less potent compared with corticosteroids, especially during acute flare in DED [7, 8, 11]. Long-term application of topical corticosteroids improved keratoepitheliopathy and tear secretion in severe inflammatory DED despite the aforementioned potential adverse effects [12]. Therefore, it is essential to discover a safe and potent anti-inflammatory agent as an alternative to topical corticosteroids.

Recently, we demonstrated that topical RCI001 effectively controlled ocular surface inflammation in several ocular surface inflammatory experimental models [13–15]. RCI001 is a novel therapeutic candidate for treating ocular surface diseases including DED. RCI001 acts as a Rac1 inhibitor and can suppress the NOD-like receptor protein (NLRP) inflammasome/ IL-1β axis, which is known as the main trigger of inflammation [15]. The active ingredient of RCI001 is 8-oxo-2ʹ-deoxyguanosine (8-oxo-dG), but its efficacy has not yet been investigated using an environmental DED model. Thus, here, we evaluated the therapeutic efficacy of RCI001 in an environmental DED mouse model and compared it with that of two commercially available topical anti-inflammatory agents for DED, 1% prednisolone acetate (PDE) and 5% Lifitegrast.

## Methods

### Ethics declarations

The protocol was approved by the Institutional Animal Care and Use Committee of Seoul National University Biomedical Research Institute (IACUC No. 20–0178-S1A0). Animal experiments were performed in accordance with the: (1) Association for Research in Vision and Ophthalmology (ARVO) statement for the use of animals in ophthalmic vision and research and (2) Animal Research: Reporting of In Vivo Experiments (ARRIVE) guidelines.

### Animals and experimental design

Twenty-four BALB/c mice (6 weeks old, female) were used. The mice were bred in a specific pathogen-free facility at the Biomedical Research Institute of Seoul National University Hospital (Seoul, Korea), maintained at 22–24 °C with 25% or less relative humidity to produce an environmental dry eye model, and had free access to food and water.

A scopolamine patch was attached to the base of the tail of the BALB/c mice. The patch was replaced every 2 days and attached for a total of 10 days, and the humidity was also kept below 25% for 17 days throughout the experiments (Supplementary Fig. 1). The patch included 1.5 mg of scopolamine (Kimite patch; Myungmoon Pharm Co., Seoul, Korea). No direct air flow was applied to the eyes of the mice. The mice were randomly divided into four groups of six mice each: phosphate-buffered saline (PBS), PDE ophthalmic suspension 1% (10 mg/mL PDE, Pred forte, Allergan, Irvine, CA), Lifitegrast ophthalmic suspension 5% (Xiidra, Novartis, AG Pharma, Basel, Swiss), and RCI001 (10 mg/mL, Rudacure, Korea). RCI001 (RCI) was dissolved in PBS. Eye drops were instilled twice daily for 7 days.

### Clinical evaluation of the dry eye

Corneal staining and tear secretion tests were performed under anesthesia (a mixture of zoletil and xylazine at a ratio of 1:3). Anesthesia was performed by intramuscular injection of tiletamine and zolazepam (30 mg/kg; Zoletil 50; Virbac, Carros, France) and xylazine hydrochloride (5 mg/kg). Ocular surface staining (OSS) scores were blindly assigned by two experienced ophthalmologists (Y.J. and J.M.) using the National Eye Institute (NEI) scoring scheme. Lissamine Green B (3%) (Sigma-Aldrich) was used for corneal staining [16, 17]. After placing one drop of dye on the conjunctival sac for 30 s, the ocular surface was gently washed with 1 mL of normal saline. Corneal staining was observed under a microscope (Olympus SZ61; Olympus, Tokyo, Japan). The mice were observed using white light (LED) illumination [16, 17]. For the tear secretion test, phenol red-impregnated cotton threads (FCI Ophthalmics, Pembroke, MA) were placed in the lateral canthus of mice for 60 s. A tear secretion test was performed after observation of OSS.

### Quantitative real-time polymerase chain reaction

The conjunctiva and cornea (CC) and extraorbital lacrimal gland (LG) were cut into small pieces and lysed in RNA isolation reagent. After sonication with a probe sonicator (Ultrasonic Processor, Cole Parmer Instruments, Vernon Hills, Illinois, USA), total RNA was extracted using the RNeasy Mini Kit (Qiagen, Venlo, Netherlands), and first-strand cDNA was synthesized by reverse transcription (High Capacity RNA-to-cDNA Kit, Applied Biosystems, Foster City, CA, USA). Real-time amplification was performed using TaqMan Universal polymerase chain reaction (PCR) Master Mix (Applied Biosystems) in an automated instrument (ABI 7500 Real-Time PCR System, Applied Biosystems) targeting tumor necrosis factor (TNF)-α (TaqMan Gene Expression Assays ID, Mm00443260_g1), interferon (IFN)-γ, IL-1β (Mm00434228_m1), IL-6 (Mm00446190_m1), IL-17a (Mm00439618_m1), IL-18 (Mm00434226_m1), and C-X-C motif chemokine ligand 1 (CXCL1) (Mm04207460_m1) in the CC. Transforming growth factor (TGF)-β (Mm01178820_m1) and IL-10 (Mm01288386_m1), including the above factors, were also assessed in the LG. Naïve mice were used as a negative control.

### Immunofluorescence staining

CC from sacrificed recipients were subjected to immunofluorescence staining. Oxidative stress induced by desiccation was assessed by immunohistochemical detection of 4-hydroxy-2-nonenal (4-HNE; a late-phase oxidative stress marker), Nicotinamide adenine dinucleotide phosphate (NADPH) oxidase 2 (NOX2), and NADPH oxidase 4 (NOX4) protein adducts [18]. The avidin–biotin-peroxidase complex (ABC) method was used for immunofluorescence staining. Tissues were fixed overnight in a 4% buffered paraformaldehyde solution and processed for paraffin embedding. Sections 4 µm thick were cut from paraffin wax blocks, mounted on precoated glass slides, deparaffinized, and rehydrated. Mean fluorescein intensity (MFI) was measured in three regions of interest (ROIs) of the conjunctival fornix and cornea using ImageJ software (National Institutes of Health, Bethesda, MD, USA).

### Statistical analyses

For between- and intra-group comparisons, Mann–Whitney U and Wilcoxon signed-rank tests were used. One-way ANOVA followed by Tukey’s multiple comparison test was used for comparison of the four groups. Results are expressed as mean ± standard deviation (SD). Data were analyzed using the GraphPad Prism software (version 9.0.1; GraphPad Software, San Diego, CA, USA). All statistical tests were performed using two-tailed tests, and *P*-values less than 0.05 were considered statistically significant.

## Results

### RCI001 significantly improved keratoepitheliopathy and tear secretion

**Fig. 1:**
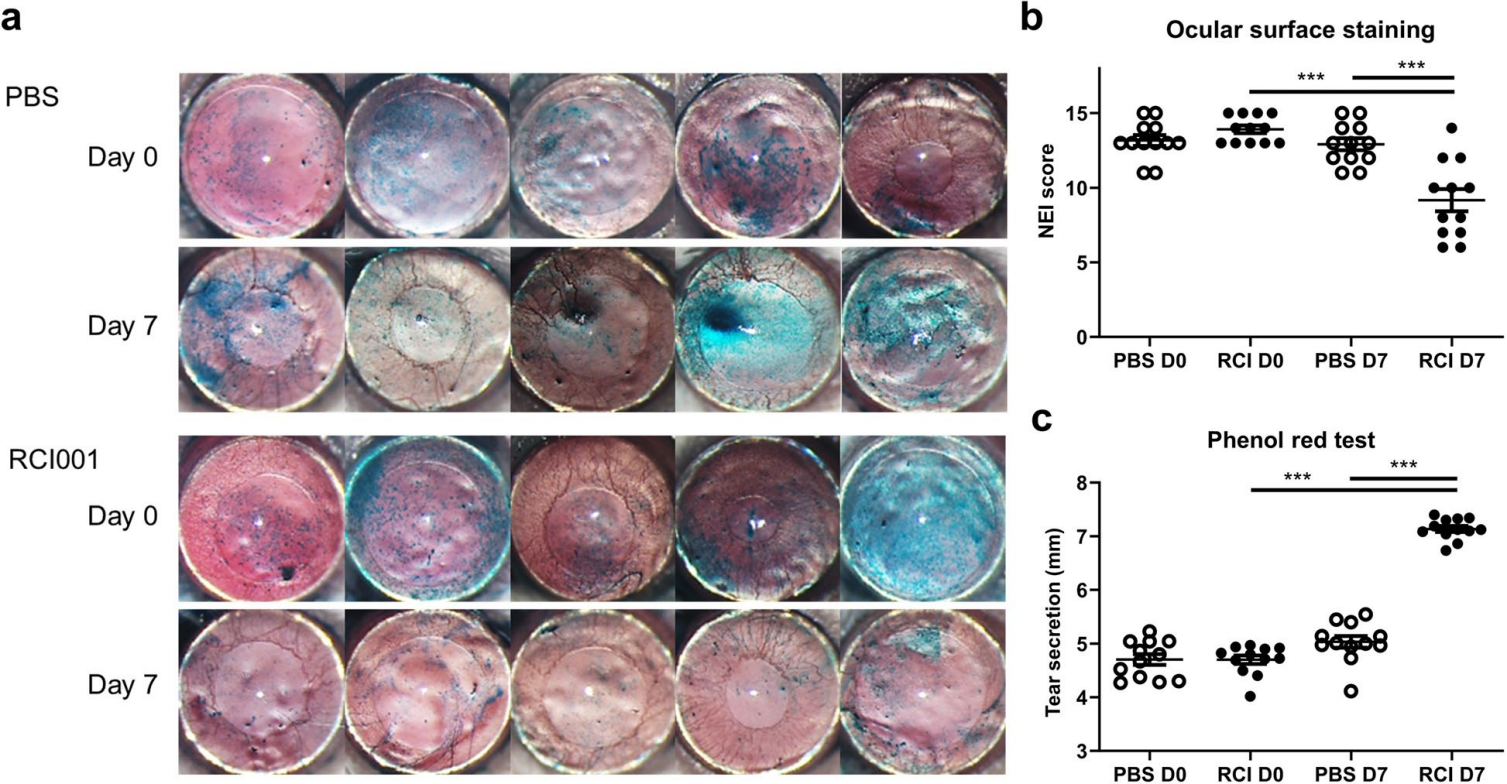
Comparison of ocular surface staining and tear secretion before and after treatment with RCI001. **a** Representative images of ocular surface staining of BALB/c mice. **b** NEI corneal staining score was significantly lower in the RCI001 group than in the PBS group (*P* < 0.01). NEI corneal score improved significantly in the RCI001 group on Day 7 (*P* < 0.01). **c** Tear secretion was significantly increased in the RCI001 group on Day 7 (*P* < 0.001) and tear secretion rate was significantly higher in the RCI001 group than in the PBS group on Day 7 (*P* < 0.001). D, day; NEI, National Eye Institute; PBS, phosphate buffered saline; RCI, RCI001. Data are expressed as mean ± standard error of the mean. ***P < 0.001, Mann–Whitney U and Wilcoxon signed-rank tests, n = 12 per group

On Day 7, the average OSS scores of RCI001 group were significantly lower (9.4 ± 3.0) than those of PBS group (13.0 ± 1.6) (*P* < 0.010; Fig. 1a–b). Tear secretion was significantly higher in the RCI001 group (7.0 ± 0.3 mm) than in the PBS group (5.0 ± 0.4 mm) (*P* < 0.001; Fig. 1c). In RCI group, the OSS score and tear secretion were significantly improved even when compared with the pre-treatment stage (Day 0 vs. Day 7, OSS score: 13.9 ± 1.1 vs. 9.4 ± 3.0; Tear secretion: 4.7 ± 0.3 mm vs. 7.0 ± 0.3 mm, *P* < 0.001, respectively; Fig. 1b–c).

### RCI001 showed better epithelial healing effects than PDE and Lifitegrast

**Fig. 2:**
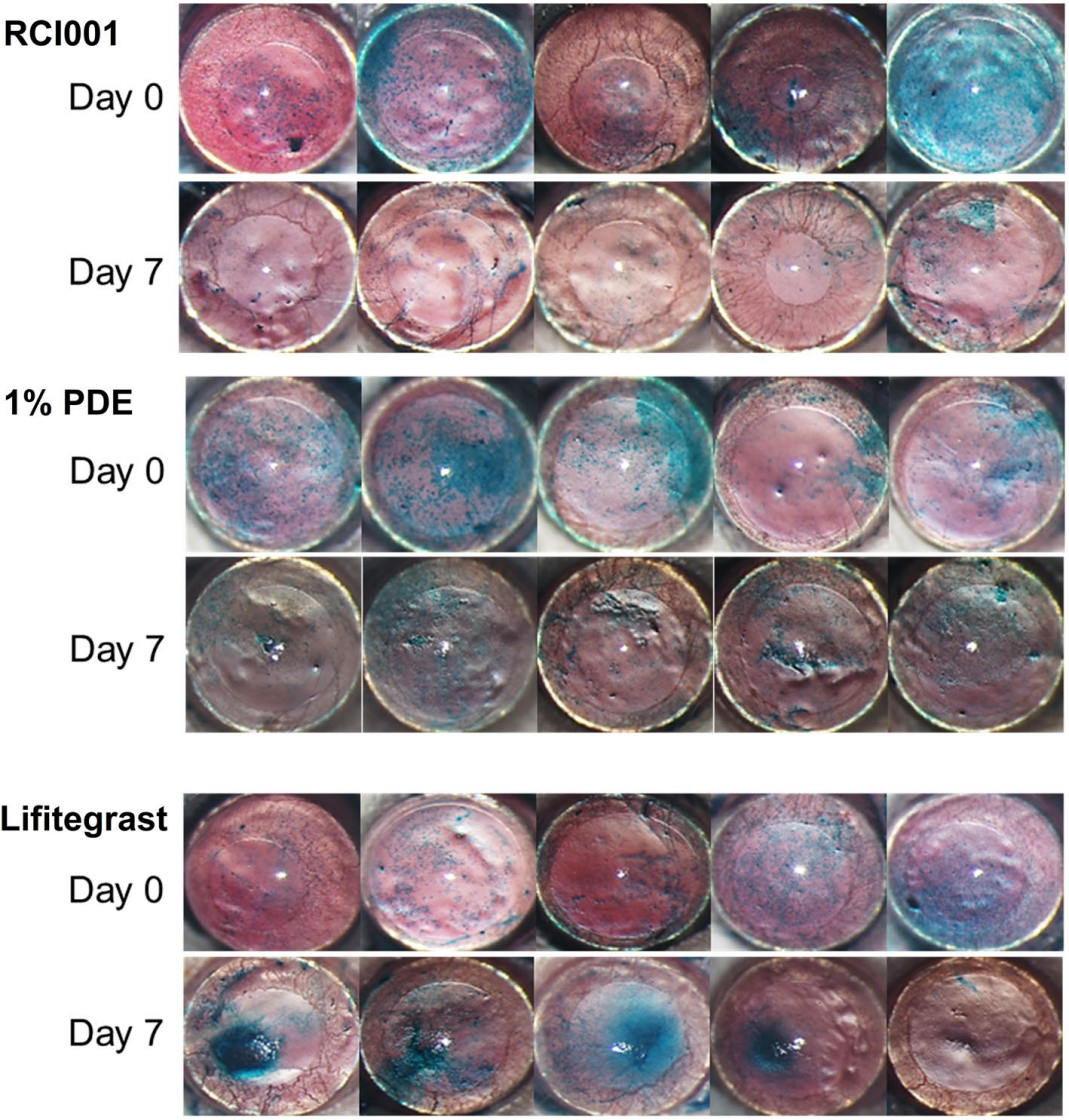
Representative images of ocular surface staining of BALB/c mice in the RCI001, 1% prednisolone acetate (PDE), and 5% Lifitegrast groups

**Fig. 3:**
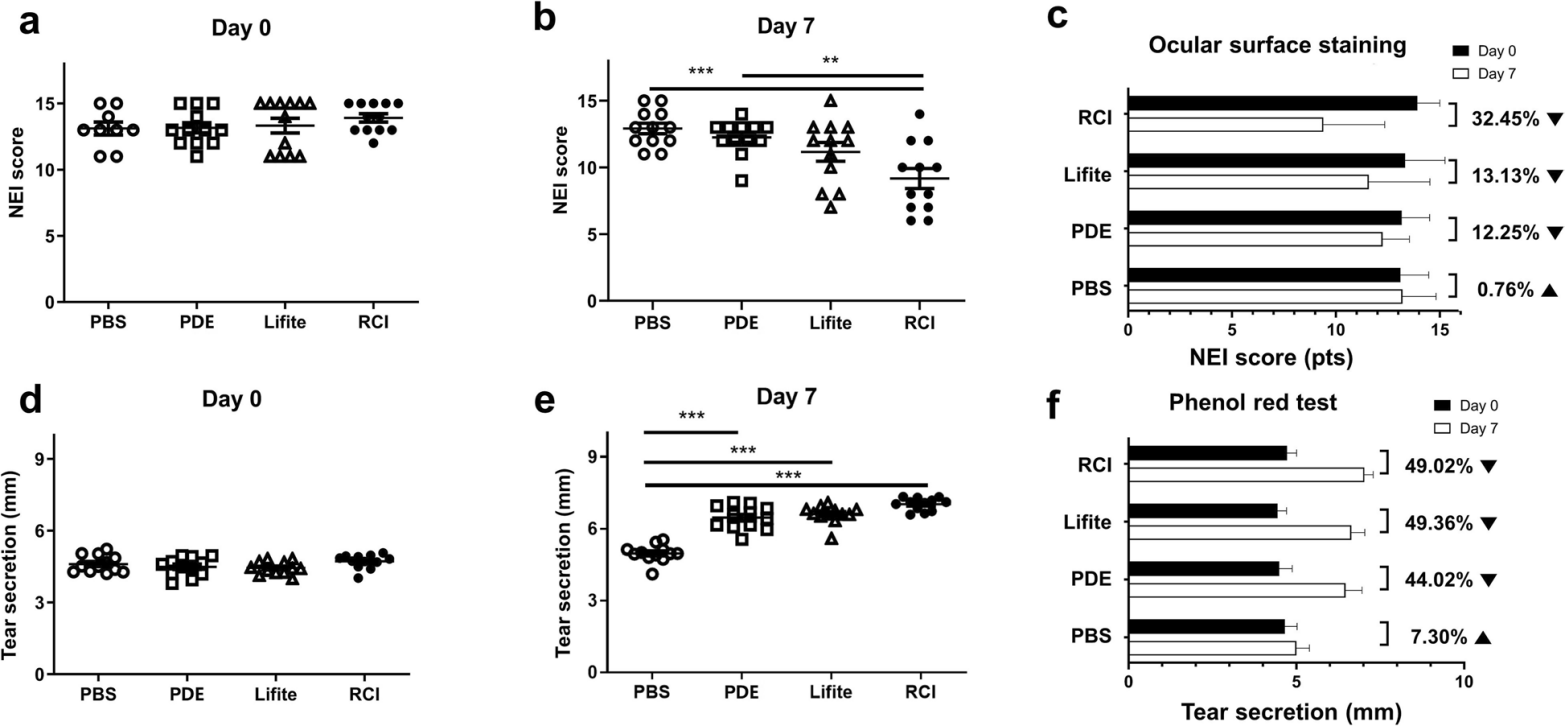
Comparison of clinical scores between the PBS, PDE, Lifitegrast, and RCI groups. **a** There was no significant difference in NEI corneal staining score of BALB/c mice at baseline between the four groups. **b** NEI corneal staining score was significantly lower in the RCI group than in the PDE group (*P* < 0.01) on Day 7. **c** Rate of change in corneal staining score between Days 0 and 7 of each group. **d** There was no significant difference in tear secretion of BALB/c mice at baseline between the four groups. **e** Tear secretion was significantly improved in the RCI, Lifitegrast, and PDE groups compared with that in the PBS group on Day 7 (*P* < 0.01). **f** Rate of change in tear secretion between Days 0 and 7 of each group. NEI, National Eye Institute; PBS, phosphate buffered saline; PDE, 1% prednisolone acetate; Lifite, 5% Lifitegrast; RCI, RCI001. Data are expressed as mean ± standard error of the mean. **P < 0.01, ***P < 0.001, one-way ANOVA with Tukey’s post hoc test, n = 12 per group

OSS scores dramatically improved in the RCI001 group on Day 7 compared to Day 0 (Fig. 2). Based on clinical photographs, the recovery of OSS in the RCI001 group seemed more effective than that in the PDE and Lifitegrast groups (Fig. 2). In the clinical score analysis, the RCI group had a significantly reduced OSS score (9.4 ± 3.0) compared with the PDE group (12.3 ± 1.3, *P* < 0.010) and comparable results to those of the 5% Lifitegrast group (10.7 ± 3.5, *P* > 0.050) on Day 7 (Fig. 3a–b). Comparing the improvement rate of OSS scores between Days 0 and 7, the RCI group showed the highest improvement (32.45%), followed by the Lifitegrast (13.13%), PDE (12.25%), and PBS (0.76%) groups (Fig. 3c).

Tear secretion was significantly increased in the RCI, PDE, and Lifitegrast groups compared to the PBS group (each *P* < 0.001, Fig. 3d–f). Among RCI, PDE, and Lifite groups, tear secretion was similar (RCI: 7.0 ± 0.3 mm, PDE: 6.5 ± 0.5 mm, and Lifite: 6.6 ± 0.4 mm). The increased tear secretion rates on Day 7 were similar between the RCI (49.02%), Lifitegrast (49.36%), and PDE (44.02%) groups. The increase in tear secretion rate in the PBS group was 7.30% (Fig. 3f).

### Inflammatory cytokine expression of the RCI group was comparable to that of the PDE, and Lifitegrast groups

**Fig. 4:**
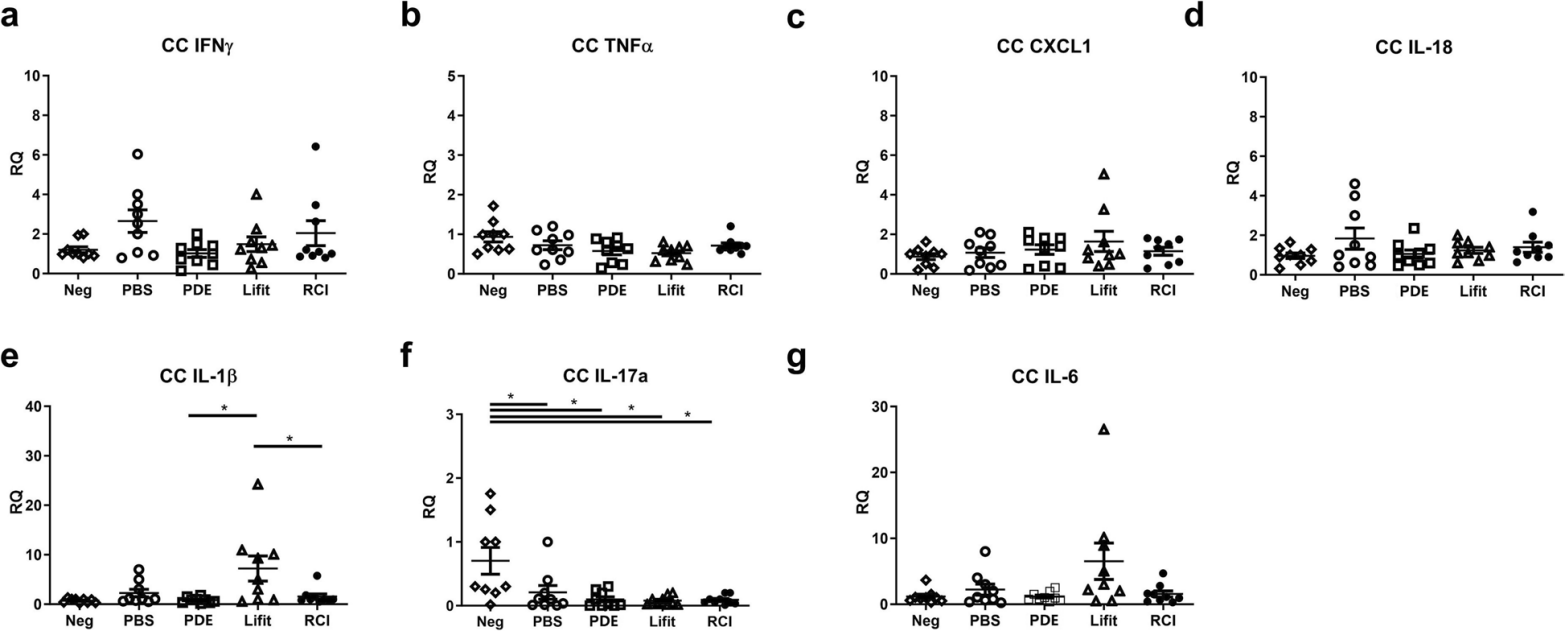
Comparison of inflammatory cytokines in the corneoconjunctiva (CC) between the Neg, PBS, PDE, Lifitegrast, and RCI groups (**a**–**g**). Neg, naïve control; PBS, phosphate buffered saline; PDE, 1% prednisolone acetate; Lifite, 5% Lifitegrast; RCI, RCI001. Data are expressed as the mean ± standard error of the mean. *P < 0.05, one-way ANOVA with Tukey’s post hoc test, n = 9 per group.) RQ, relative quantification of mRNA expression

**Fig. 5:**
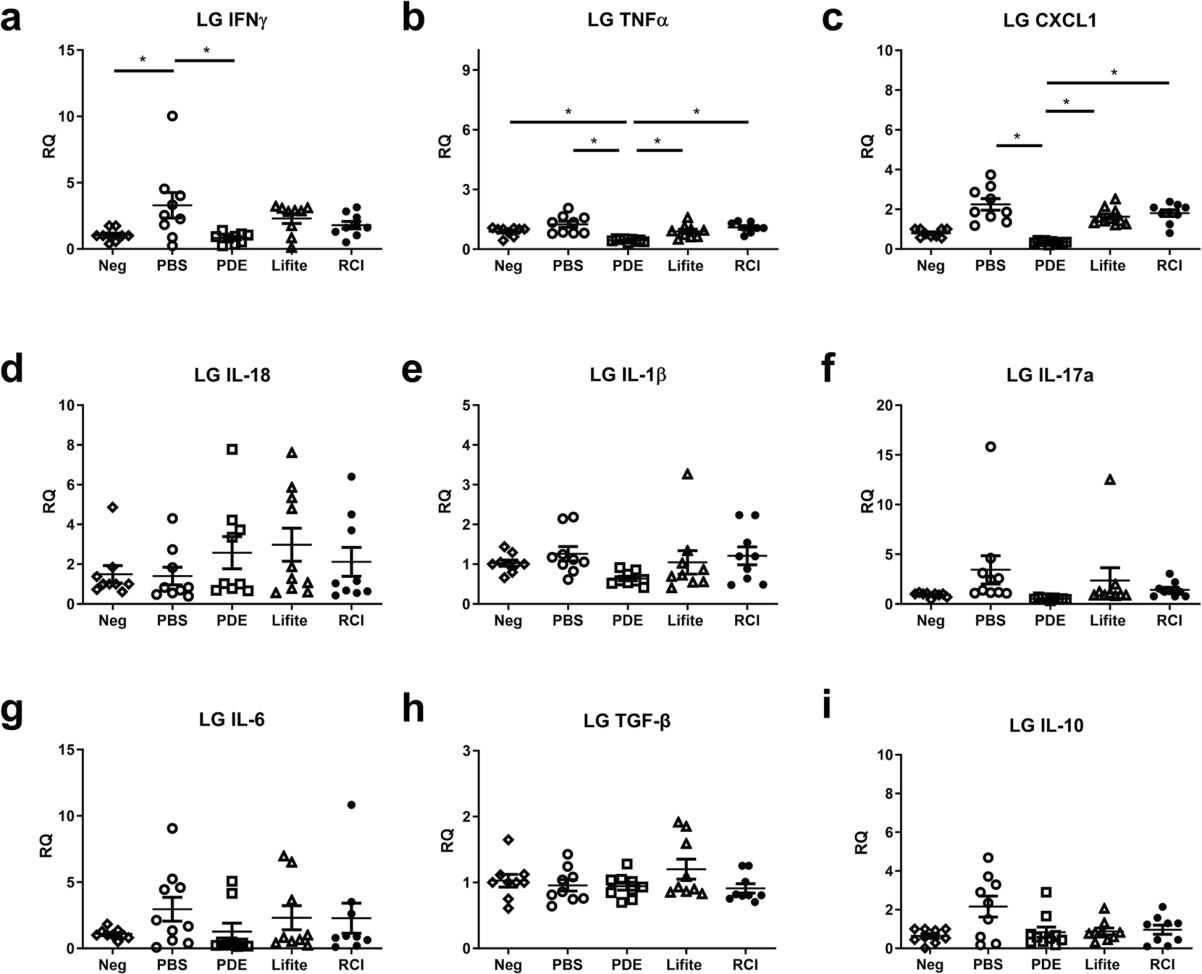
Comparison of inflammatory cytokines in the lacrimal gland (LG) between the Neg, PBS, PDE, Lifitegrast, and RCI groups (**A**–**I**). Neg, naïve control; PBS, phosphate buffered saline; PDE, 1% prednisolone acetate; Lifite, 5% Lifitegrast; RCI, RCI001. Data are expressed as the mean ± standard error of the mean. *P < 0.05, one-way ANOVA with Tukey’s post hoc test, n = 9 in each group. RQ, relative quantification of mRNA expression

In the RCI group, most inflammatory cytokines in the CC (IFN-γ, TNF-α, CXCL1, IL-18, IL-1β, and IL-6) and LG (IFN-γ, TNF-α, CXCL1, IL-18, IL-17a, IL-1β, IL-6, TGF-β, and IL-10) were not significantly changed compared to the naïve control (Neg) and Lifitegrast groups (*P* > 0.050, Figs. 4 and 5). Additionally, there were no significant differences in inflammatory cytokines of CC between the RCI and PDE groups except for IFN-γ in CC and the difference of IFN-γ levels were slight (Fig. 4a). IL-17a levels of CC in the Neg group were slightly increased compared with those of other treatment groups (PBS, PDE, Lifitegrast, and RCI) (*P* < 0.050, Fig. 4). The PDE group showed lower expression of TNF-α and CXCL1 in the LG than the Lifitegrast and RCI groups; however, the differences were slight (Fig. 5b, c).

### RCI treatment resulted in decreased levels of oxidative stress markers

**Fig. 6:**
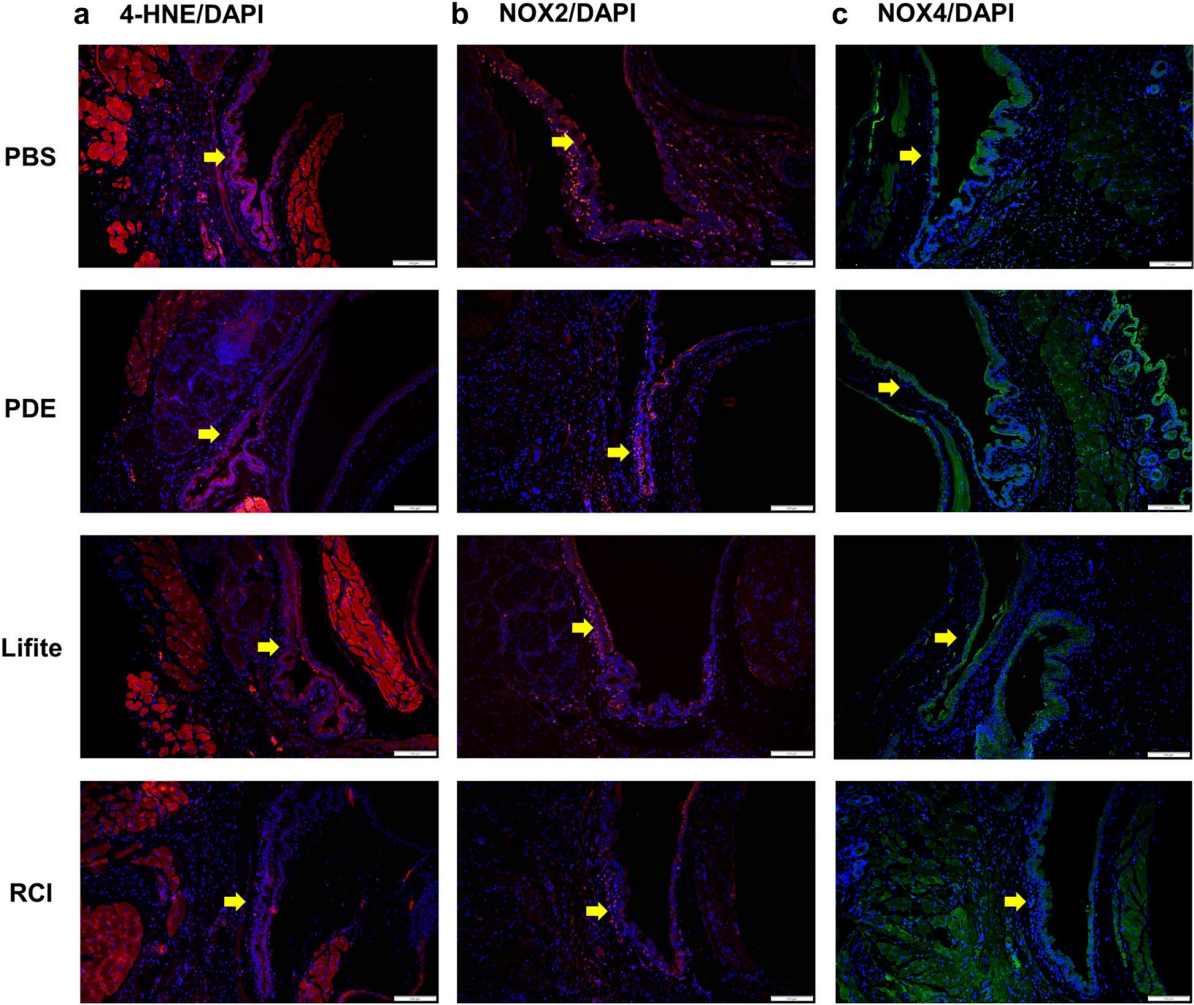
Representative images of the immunofluorescence staining (200 ×) of 4-HNE (**a**), NOX2 (**b**), and NOX4 (**c**) expression in conjunctiva dissected from the experimental dry eye model. The RCI group saw reduced expression levels of 4-HNE, NOX2, and NOX4, and the decrease was comparable to that in the PBS, PDE, and Lifitegrast groups (yellow arrows). Red, 4-HNE and NOX2; green, NOX4; blue, nuclear DAPI staining. Scale bar: 100 μm, n = 3 per group; PBS, phosphate buffered saline; PDE, 1% prednisolone acetate; Lifite, 5% Lifitegrast; RCI, 1.0% RCI001; 4-HNE, 4-hydroxy-2-nonenal; NOX2, nicotinamide adenine dinucleotide phosphate oxidase 2; NOX4, nicotinamide adenine dinucleotide phosphate oxidase 4; DAPI, 4′,6-diamidino-2-phenylindole

**Fig. 7:**
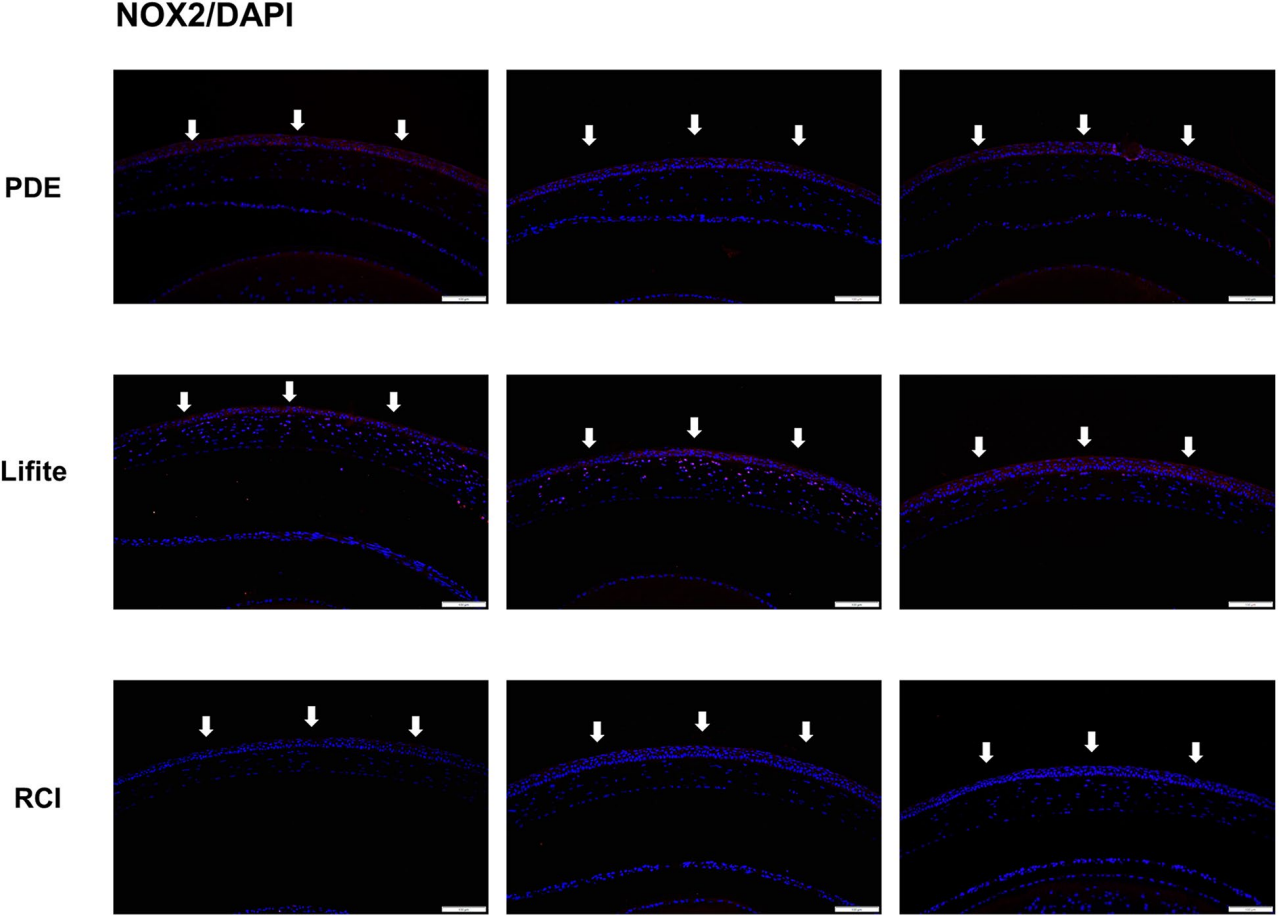
Representative images of the immunofluorescence staining (200 ×) of NOX2 expression in the cornea dissected from the experimental dry eye model. The RCI group showed reduced expression of NOX2, and the reduction was comparable to that in the PDE and Lifitegrast groups (white arrows). Red, NOX2; blue, nuclear DAPI staining. Scale bar: 100 μm; n = 3 per group. PDE, 1% prednisolone acetate; Lifite, 5% Lifitegrast; RCI, 1.0% RCI001; NOX2, nicotinamide adenine dinucleotide phosphate oxidase 2; DAPI, 4′,6-diamidino-2-phenylindole

**Fig. 8:**
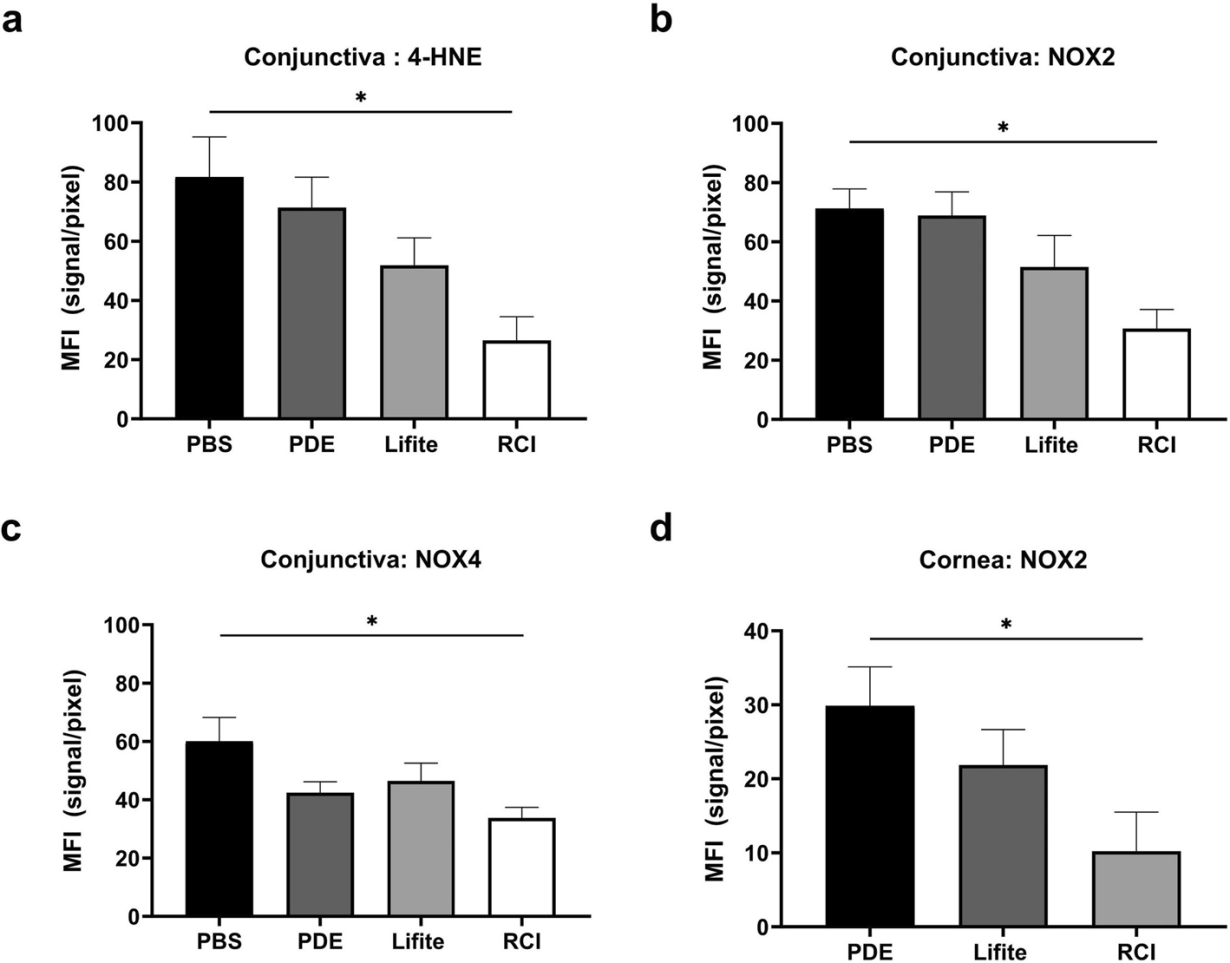
Mean fluorescein intensity (MFI) values of 4-HNE, NOX2, and NOX4 of conjunctiva (**a**–**c**) and those of NOX2 of cornea (**d**). The MFI values of RCI group in the CC were only significantly decreased compared to those of PBS group. PBS, phosphate buffered saline; PDE, 1% prednisolone acetate; Lifite, 5% Lifitegrast; RCI, RCI001; 4-HNE, 4-hydroxy-2-nonenal; NOX2, nicotinamide adenine dinucleotide phosphate oxidase 2; NOX4, nicotinamide adenine dinucleotide phosphate oxidase 4; DAPI, 4′,6-diamidino-2-phenylindole; n = 3 per group. Data are expressed as the mean ± standard error of the mean. *P < 0.05, one-way ANOVA with Tukey’s post hoc test

The RCI group showed notably weaker 4-HNE, NOX2 (red), and NOX4 fluorescence (green) than the PBS, PDE, and Lifitegrast groups in the conjunctival fornix (yellow arrow, Fig. 6). Additionally, the RCI group showed weaker red fluorescence of NOX2 in the cornea than the PDE and Lifitegrast groups (white arrow, Fig. 7). In the conjunctiva, the MFI values of 4-HNE, NOX2, and NOX4 of the RCI group were significantly lower than those of the PBS group (each *P* < 0.050, Fig. 8a–c). Additionally in the cornea, NOX2 in the RCI group was significantly lower than that of the PDE group (*P* < 0.050, Fig. 8d).

## Discussion

Here, the RCI group demonstrated excellent therapeutic effects compared to the PBS group, and the effects of RCI were comparable with those of 1% PDE and 5% Lifitegrast in clinical and molecular biological aspects in the environmental DED model. In particular, corneal epithelial healing effects and suppression of oxidative stress on the ocular surface in the RCI group were better than those in the PDE and Lifitegrast groups. The PDE group showed the best suppression of inflammatory cytokines in the CC and LG; however, the difference was slight compared to the RCI group.

8-oxo-dG is a substance that is released when the guanine base of cellular DNA is damaged [19]. Interestingly, exogenous applications of 8-oxo-dG showed potent anti-inflammatory and anti-oxidative effects in several inflammatory disease models through Rac1 inhibition [19–22]. Rac1-associated functions include phagocytosis, chemotaxis, inflammatory cytokine release, and reactive oxygen species production through NADPH oxidase activation [23]. Additionally, Rac1 is associated with the regulation of mitogen-activated protein kinase (MAPK), extracellular signal-regulated kinase (ERK), Janus kinase (JAK)/signal transducer and activator of transcription (STAT), and nuclear factor kappa light chain enhancer of activated B cells (NF-κB) [23–25]. Our previous study showed that topical RCI001 suppressed activation of neutrophils and macrophages and inhibited Rac1/NLRP3 inflammasome/IL-1β axis in experimental ethanol injury and alkali burn models [13–15]. Furthermore, expression of TNF-α, NOX2, and NOX4 after RCI001 treatment was significantly lower compared with that after PDE treatment in an alkali-burned cornea mouse model [14].

The immune component functioning at the ocular surface involves both innate and adaptive systems [26]. Complex regulatory processes protect the ocular surface; however, when dysregulated, it can lead to DED [5, 27]. Various environmental stresses such as wind, low humidity, air pollution, and video display terminals usage trigger an innate immune response of DED on the ocular surface [5, 28, 29], and MAPK, ERK, and NF-κB can be induced by this activated innate response [8]. A variety of immune cells, cytokines, and chemokines are involved in this complex immune pathway of DED: dendritic cells (antigen-presenting cells), neutrophils, macrophages, T cells (CD4^+^ and CD8^+^), TNF- α, IL-1, IL-6, IL-17, IFN-γ, CCR7, and CXCL1 [30]. Topical agents for DED that are currently available include corticosteroids, cyclosporine, Lifitegrast, diquafosol, and rebamipide. These agents are involved in complex DED immunopathogenic pathways and exert therapeutic effects through various mechanisms of action. However, there are evident unmet medical needs with regard to therapeutics for DED owing to limitations of long-term usage, irritation, and insufficient clinical effects. RCI is thought of as a promising candidate for DED therapeutics. RCI can act as a powerful inhibitor of multiple inflammatory mediators and various immune cells, similar to corticosteroids, and has potent antioxidative effects [14, 15]. Our preclinical in vivo study also demonstrated that long-term topical application of RCI001 for more than 5 weeks did not induce elevation of intraocular pressure [31]. In accordance with the results of our previous study, RCI showed better epithelial healing and suppression of oxidative stress than 1% PDE and 5% Lifitegrast [13–15]. Inflammatory cytokines were not highly activated in the environmental DED model in this study, and the PDE group was the most effective in suppressing inflammatory cytokines. PDE is the most potent topical corticosteroid and Lifitegrast is a novel integrin antagonist which prevents LFA-1/ICAM-1 interaction preventing T-cell activation/recruitment and release of inflammatory mediators. However, Lifitegrast is not available in South Korea at the moment. Therefore, we compared these agents with RCI. Nevertheless, given the greater improvement in OSS and tear secretion in the RCI group than in the PDE group, the anti-oxidative potency of RCI may suppress different pathways, unlike corticosteroids. We will continue to verify the efficacy of RCI in the inflammatory DED model (Primary Sjogren syndrome model) and other ocular surface inflammatory disease models.

This study had several limitations. First, the sample size was small. Second, the long-term changes or more environmental ocular stress were not assessed in the experimental models. Third, meibomian gland dysfunction, which is a major cause of DED, was not evaluated. Nevertheless, this study demonstrated that RCI has an excellent anti-inflammatory and antioxidative effects comparable to those of corticosteroids and Lifitegrast.

## Conclusion

This study revealed that topical RCI effectively improved keratoepitheliopathy and tear secretion, and its efficacy was better than that of the commercially available 1% PDE and 5% Lifitegrast in environmental DED models. RCI also effectively suppressed oxidative stress on the ocular surface compared with the two commercially available agents. These excellent therapeutic effects of RCI in ocular surface diseases were consistent with our previous studies [13–15]. Considering the various mechanisms of action of RCI and complex immunopathogenesis in DED, we believe that topical RCI is a promising therapeutic agent for DED.

### Supplementary Information

Supplementary Material 1: Supplementary Fig. 1. Experimental protocols.

## Data Availability

The data that support the findings of this study are available from the corresponding author upon reasonable request.
