# Supplementary figures and images for: Efficacy of RCI001 as a therapeutic candidate of dry eye disease in a modified mixed dry eye model
Source: Eye Vis (Lond). 2024 Jun 1;11:19. doi: 10.1186/s40662-024-00388-z (PMC11143567; doi:10.1186/s40662-024-00388-z)

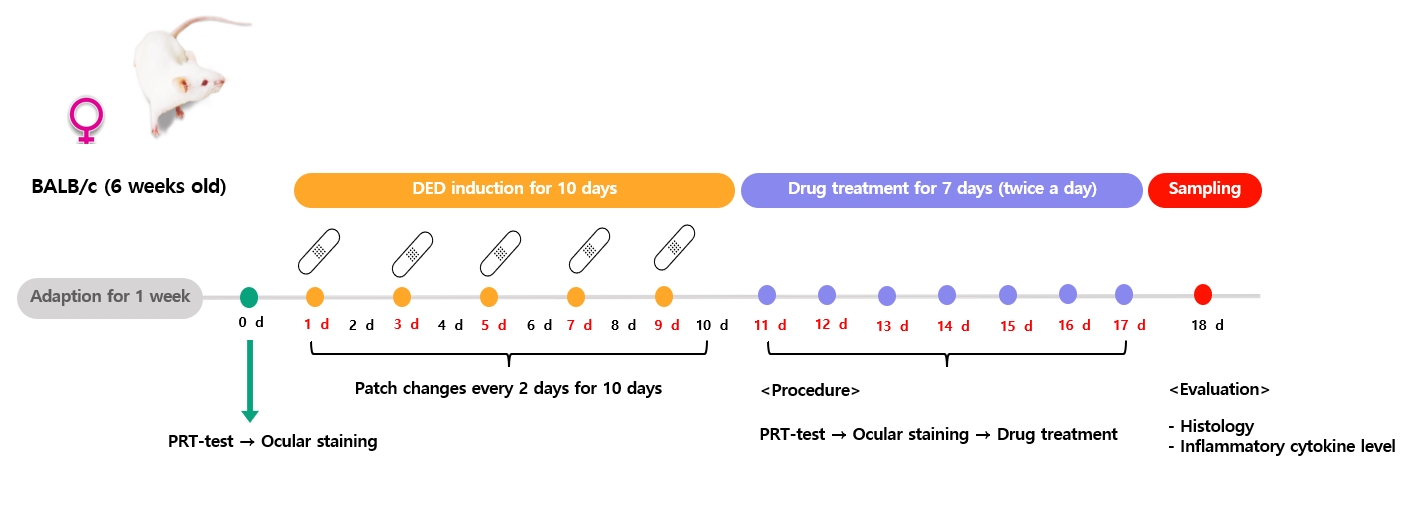

Supplement: Supplementary file 1 — Supplementary Material 1: Supplementary Fig. 1. Experimental protocols. [file 40662_2024_388_MOESM1_ESM.jpg]
